# Characterization of intraocular pressure responses of the Tibetan monkey (*Macaca thibetana*)

**Published:** 2011-05-27

**Authors:** Guo Liu, Tao Zeng, Wenhan Yu, Naihong Yan, Hongxing Wang, Su-ping Cai, Iok-Hou Pang, Xuyang Liu

**Affiliations:** 1 Ophthalmic Laboratories & Department of Ophthalmology, West China Hospital, Sichuan University, Chengdu, Sichuan, P.R. China; 2 State Key Laboratory of Biotherapy, Sichuan University, Chengdu, Sichuan, P.R. China; 3 Institute of Laboratory Animal Science, Sichuan Academy of Medical Sciences, Chengdu, Sichuan, P.R. China; 4 Glaucoma Research, Alcon Research Ltd., Fort Worth, TX

## Abstract

**Purpose:**

To characterize the effects of circadian rhythm, feeding time, age, general anesthesia, and ocular hypotensive compounds on intraocular pressure (IOP) of the Tibetan monkey (*Macaca thibetana*).

**Methods:**

Tibetan monkeys were trained for IOP measurement with the TonoVet® rebound tonometer without sedation or anesthesia. Their circadian IOP fluctuation was monitored every 3 h. Effects of changing the feeding time, general anesthesia, age (2–3 year-old versus 8–15 year-old animals), and various pharmacological agents, such as travoprost, timolol, naphazoline and spiradoline, on IOP were also evaluated.

**Results:**

After behavioral training, conscious Tibetan monkeys were receptive to IOP measurement. The lowest and highest IOP values in a circadian cycle were recorded at 3:00 AM (19.8±0.4 mmHg, mean±SEM, n=12) and noon (29.3±0.9 mmHg), respectively. Changing the feeding time from 11:30 AM to 12:30 PM lowered the noon IOP to 25.1±1.2 mmHg. General anesthesia lowered IOP in these monkeys, while IOP of young and mature animals were similar. Three hours after topical ocular administration, travoprost reduced IOP by 5.2±0.6 mmHg (n=6, p<0.001), and timolol reduced IOP by 2.8±0.7 mmHg (p<0.05). Naphazoline and spiradoline lowered IOP by 4.8 mmHg and 2.5 mmHg (both p<0.001), respectively, 2 h after drug administration.

**Conclusions:**

The circadian IOP fluctuation in conscious Tibetan monkeys and their responses to travoprost, timolol, and other experimental conditions are similar to other primates. These monkeys appear to be a suitable model for glaucoma research.

## Introduction

Glaucoma is one of the leading causes of irreversible vision loss worldwide, estimated to affect 80 million by 2020 [1]. This disease is a complex, age-related, and inherited optic neuropathy with characteristic slow progressive loss of retinal ganglion cells and excavation of the optic disc. An elevated intraocular pressure (IOP) is a major risk factor. Many prospective and randomized clinical trials have consistently demonstrated that lowering IOP is important in slowing the progression of glaucoma, as well as preventing and delaying its onset [2-6]. Correspondingly, all currently available glaucoma treatments, be they pharmaceutical or surgical, are designed to lower IOP [7].

Animal models are widely used and critical in glaucoma research, especially for the evaluation and development of IOP-lowering therapies. Various laboratories have used rodent, rabbit, feline, canine, porcine, bovine, and ovine models for this purpose [8]. Recently, mouse models of glaucoma have become popular. Aided by the power of mouse genetics, they allow researchers to dissect pathogenic pathways responsible for glaucomatous damage to the eye at the molecular and genetic levels [9-11]. Despite the advantages of the various animal models, non-human primates are very valuable because of their anatomic and functional similarities to human in ocular structures relevant to the disease [12,13]. All approved IOP-lowering medications lower IOP in the monkey [14]. Development of novel IOP-lowering therapies also often depends on critical in vivo findings provided by the monkey model. Hence, they are highly valuable for glaucoma research. The most commonly used monkeys for IOP studies have been the cynomolgus monkeys and rhesus monkeys [15-17].

To expand the repertoire of monkey models for glaucoma research, in the current report, we describe previously un-characterized IOP responses of the Tibetan monkey (*Macaca thibetana)* under baseline and various experimental conditions. The Tibetan monkey, also known as Chinese Stump-tailed Macaque or Milne-Edwards' Macaque, is a widely distributed non-human primate in central and southwestern China, ranging as far south as the Guangxi province and as far west as the Yangtze Gorges in western and northwestern Sichuan province. These animals live in broadleaf evergreen, subtropical, and deciduous forests 1 to 2.5 km in elevation. They are predominantly diurnal and frugivorous, but also consume other parts of plants and invertebrates. Compared to rhesus monkeys *(Macaca mulatta)*, Tibetan monkeys have larger body size, longer life span (over 20 years), calmer temperament, and are easier to train [18]. They are legally approved to be used for scientific research by the State Forestry Administration in the People’s Republic of China.

In this study, we have trained Tibetan monkeys to calmly allow IOP measurement by the TonoVet® rebound tonometer without sedation or anesthesia. The circadian fluctuation of IOP and influences of general anesthesia, age, and feeding time on IOP were evaluated. Additionally, the IOP effects of clinical IOP-lowering medications, such as travoprost and timolol, as well as experimental compounds, such as adrenergic and imidazoline receptor agonist naphazoline and kappa opioid agonist spiradoline, which were reported to lower rabbit IOP [19,20], were also assessed in these monkeys. To our knowledge, this is the first report characterizing the IOP responses of conscious *Macaca thibetana.* These observations may serve as an initial foundation for the use of these animals as a model for studying glaucoma and IOP-lowering therapies.

## Methods

### Animals

Twelve young (2–3 years old) and six mature (8–15 years old) Tibetan monkeys in equal numbers of males and females were used in this study. They were purpose-bred for research use by the Institute of Laboratory Animal Sciences, Sichuan Academy of Medical Sciences (Chengdu, China). All animal experiments were conducted in compliance with the ARVO Statement for the Use of Animals in Ophthalmic and Vision Research, the Guide for the Care and Use of Laboratory Animals (National Research Council, Chengdu Sichuan), and under the supervision of the Institutional Animal Care and Use Committee in Sichuan University. The animals were housed under 12-h/12-h light/dark cycle with lights on starting at 6:00 AM Physical examinations including ophthalmic examinations were conducted in all monkeys before the experiments to exclude potential health factors that might affect IOP measurements.

### Behavioral training

**Figure 1:**
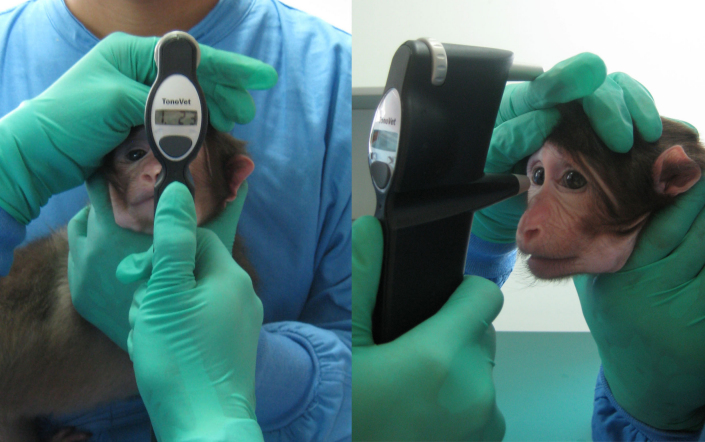
Measurement of IOP by the TonoVet® rebound tonometer in conscious Tibetan monkey. A trained monkey was gently held in an assistant's arms, allowing IOP measurement with the rebound tonometer.

For this study, only the young (2–3 years old) monkeys were trained for conscious IOP measurement. The mature (8–15 years old) animals were not trained and used only for the anesthesia effect study. Behavioral training was conducted based on the principle of award-conditioned behavior. Newborn monkeys were raised in the laboratory, with daily close and friendly interactions with caretakers and trainers. Training for IOP measurement started at the age of two. Two trainers participated in the training, one holding the monkey gently in his arms, and the other one using the tonometer to measure IOP. The animal was gently restrained but not stressed (Figure 1). If the monkey cooperated, a peanut was given as a reward, which was one of the monkey’s favorite foods but not provided in its daily food ration. After a five- to six-month daily training period, all monkeys showed good tolerance to the tonometer and appeared calm and comfortable during IOP measurement. Consistent and reproducible IOP values were easily obtained from these animals.

### IOP measurement

IOP was measured using the TonoVet® rebound tonometer (Tiolat Oy, Helsinki, Finland) according to the manufacturer’s recommended procedures. We have previously shown that the equipment reports actual monkey IOP accurately and reproducibly [13]. The tonometer was programmed to calculate and display the mean IOP value of six consecutive, acceptable measurements. In this study, six mean values were obtained from each eye, at each time point, under each condition, and the mean of means treated as a single datum. All IOP measurements were conducted in the same room at room temperature, with humidity approximately 70%. Illumination intensity was set at 200 lx during the day (6 AM to 6 PM) and 10 lx at night (6 PM to 6 AM).

### Circadian IOP fluctuation study

The 24-h IOP fluctuation measurements were conducted in two studies, one predominantly diurnal and the other predominantly nocturnal. In the diurnal study, IOP was measured every 3 h, at 9:00 AM, noon, 3:00 PM, 6:00 PM (immediately before lights off), and 9:00 PM in the same day. In the nocturnal study, IOP measurement was performed 3 days later, at 9:00 PM, mid-night, 3:00 AM, 6:00 AM (immediately before lights on), and 9:00 AM

### Effect of feeding time on IOP

The monkeys were routinely fed at 11:30 AM To evaluate the effect of this feeding time on the IOP measurement at noon, feeding time was changed to 12:30 PM for a week, and IOP was measured at noon before and one week after the change.

### Effect of anesthesia and age on IOP of monkeys

Six mature (8–15 years old) monkeys and 6 young (2–3 years old) monkeys, half males and half females, were used in this study. At noon and at 3:00 AM, animals were anesthetized by ketamine (50 mg/kg, intramuscular injection). IOP was assessed approximately 5 min after the monkeys were injected, at which time they were sufficiently anesthetized to allow measurement.

### Drug effects on IOP

Effects of four compounds were evaluated in four separate studies. In each study, trained animals were randomized and divided into two groups. One group was treated with topical ocular administration (30 μL) of BSS® solution (Alcon Laboratories, Fort Worth, TX) as vehicle control. The other group was treated with one of the following: travoprost (0.004%, Travatan®; Alcon Laboratories), timolol (0.5%; Qingshan Pharmaceuticals Company, Chengdu, China), naphazoline (0.33% in 0.9% saline; Sigma, St. Louis, MO), or spiradoline (0.33% in 0.9% saline; Sigma). IOP measurements by a researcher masked to the treatments were performed at 9 AM (travoprost and timolol) or 10 AM (naphazoline and spiradoline) immediately before drug dosing (time=0 h) and subsequent time points as indicated. A wash-out period of at least one week between drug treatments was strictly observed.

### Statistics

Data were analyzed by SPSS 17.0 statistics software (SPSS Inc., Chicago, IL). One-way ANOVA followed by Bonferroni’s test was used to compare IOP values among three or more groups. IOP values between two conditions were compared by Two-tailed paired Student’s *t*-test. Data are presented as mean ± standard error of mean (SEM). Differences are regarded as significant when p<0.05.

## Results

### Circadian IOP fluctuation

**Figure 2:**
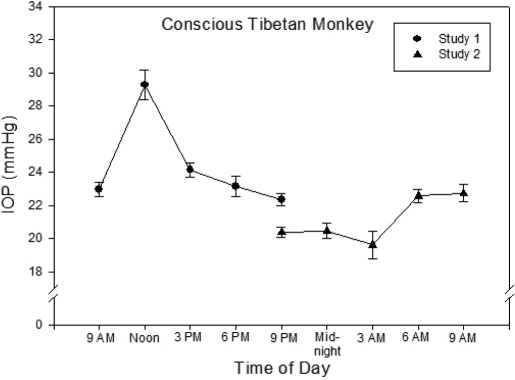
Circadian IOP fluctuation of conscious Tibetan monkeys. IOP was measured by the TonoVet® rebound tonometer at the indicated times of the day. Data of two studies conducted three days apart on the same animals are presented together: the first study monitored IOP from 9 AM to 9 PM; the second, from 9 PM to 9 AM Symbols represent mean±SEM (n=12; both eyes of 6 animals).

Circadian IOP fluctuation was clearly detected in the conscious Tibetan monkeys. In this study, measurements of IOP were conducted at 9 AM, noon, 3 PM, 6 PM (immediately before lights off), and 9 PM in the same day. Three days later, IOP of the same animals were monitored again at 9 PM, midnight, 3 AM, 6 AM (immediately before lights on), and 9 AM By combining data from these two studies, a 24-h IOP profile was obtained. As seen in Figure 2, the IOP values of Tibetan monkeys were generally higher during the day and lower at night. They were between 20 and 24 mmHg at most time points. However, a distinct elevation of IOP was observed at noon (29.3±0.9 mmHg, mean±SEM, n=12). The lowest IOP reading (19.6±0.8 mmHg) occurred at 3:00 AM (Figure 2).

### Effect of feeding time on IOP

**Figure 3:**
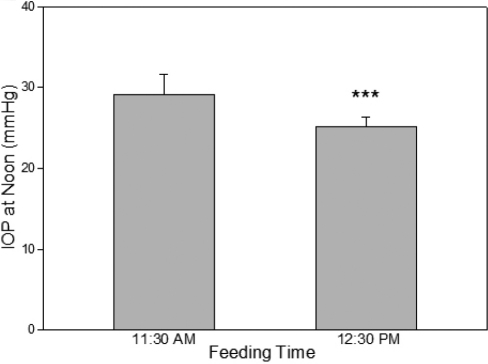
Effect of feeding time on IOP. IOP of conscious monkeys was measured at noon at two different feeding times: 11:30AM and 12:30 PM Error bars represent SEM of IOP values (n=12; both eyes of 6 animals). ***p<0.001 by paired two-tailed Student's *t*-test.

The monkeys were routinely fed at 11:30 AM We speculated that this may contribute to the elevated IOP observed at noon. To assess this hypothesis, the feeding time was changed from 11:30 AM to 12:30 PM for one week, and IOP at noon was measured, before and then one week after the feeding time change. Changing the feeding time did affect IOP. When fed at 11:30 AM, monkeys had IOP of 29.2±2.5 mmHg (n=12) at noon. Feeding at 12:30 PM lowered the noon IOP to 25.1±1.2 mmHg (Figure 3). To be compliant with the animal care policy of the institute, for all other studies in this report, animals were fed at 11:30 AM The feeding in the afternoon was conducted at 4:30 PM, which did not seem to have the ocular hypertensive effect on the 6:00 PM IOP measurement.

### Effects of anesthesia and age on IOP

Both anesthesia and age are known to affect IOP. We monitored IOP at noon and 3 AM in six young (2–3 years old) and six mature (8–15 years old) Tibetan monkeys after general anesthesia by ketamine. The values were also compared to those of young conscious monkeys at the same time points. Table 1 shows that at noon, young conscious monkeys had the highest IOP (29.3±0.9 mmHg), which was significantly higher than both anesthetized young (22.1±0.7 mmHg) and anesthetized mature monkeys (19.9±1.1 mmHg). Furthermore, the anesthetized young monkeys appeared to have a higher IOP than the anesthetized mature animals, but the difference was not statistically significant. At 3 AM, IOP was similar among the three study groups.

**Table 1:** Effects of anesthesia and age on monkey IOP.

**Age**	**State**	**n**	**IOP at Noon (mmHg)**	**IOP at 3 AM (mmHg)**
Young	Conscious	12	29.3±0.9*	19.6±0.8
Young	Anesthetized	12	22.1±0.7	19.7±0.4
Matured	Anesthetized	12	19.9±1.1	18.0±1.4

### Drug effects on IOP of conscious monkeys

**Figure 4:**
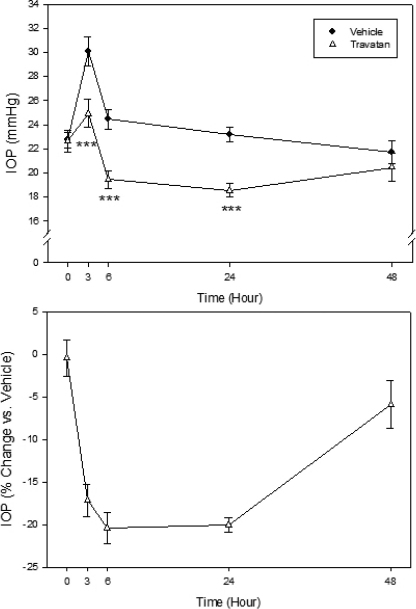
Effect of travoprost (0.004%, 30 μl) on IOP of conscious Tibetan monkeys. The drug was administered topically at 9 AM (Time 0) onto a randomly assigned eye of each animal, while the contralateral eye received vehicle as control. IOP was monitored at 3, 6, 24, and 48 h later. Error bars represent SEM (n=6). Top Panel: IOP values of both groups. ***p<0.001 between the two treatment groups by two-tailed paired Student's *t*-test. Bottom panel: % IOP change of the travoprost group relative to the vehicle control group.

**Figure 5:**
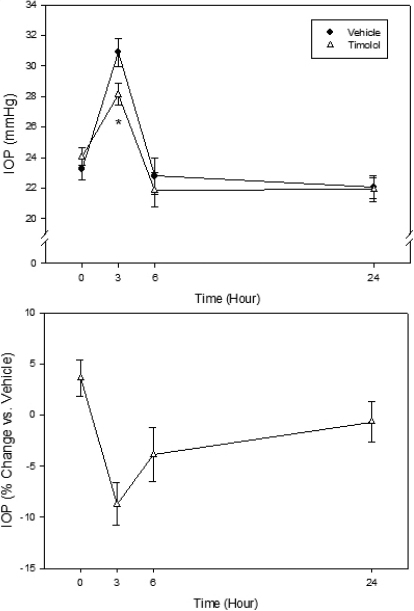
Effect of timolol (0.5%, 30 μl) on IOP of conscious Tibetan monkeys. The drug was administered topically at 9 AM (Time 0) onto a randomly assigned eye of each animal, while the contralateral eye received vehicle as control. IOP was monitored at 3, 6, and 24 h later. Error bars represent SEM (n=6). Top Panel: IOP values of both groups. *p<0.05 between the two treatment groups by two-tailed paired Student's *t*-test. Bottom panel: % IOP change of the timolol group relative to the vehicle control group.

To determine the Tibetan monkey’s IOP responses to pharmacologically active compounds, travoprost (0.004%; 30 µl) and timolol (0.5%; 30 µl), both of which are widely used ocular hypotensive drugs approved for human use, were tested in these animals. Topical ocular administration of either travoprost or timolol reduced IOP in conscious Tibetan monkeys. After drug treatment, travoprost reduced IOP by 17.2% (5.2 mmHg; p<0.001) from the vehicle group at 3 h, by 20.4% (5.0 mmHg; p<0.001) at 6 h, and by 20.0% (4.6 mmHg; p<0.001) at 24 h. No statistical difference was found between thses two group at 48 h, indicating that the IOP lowering effects of travaprost lasting less than 48 h (Figure 4). Timolol was also effective in lowering IOP in these monkeys, though much less efficacious and shorter acting than travoprost. Compared to the vehicle-treated group, timolol reduced IOP by 8.7% (2.8 mmHg; p<0.05) at 3 h after drug administration. At 6 h and 24 h, no significant IOP effect was observed (Figure 5).

**Figure 6:**
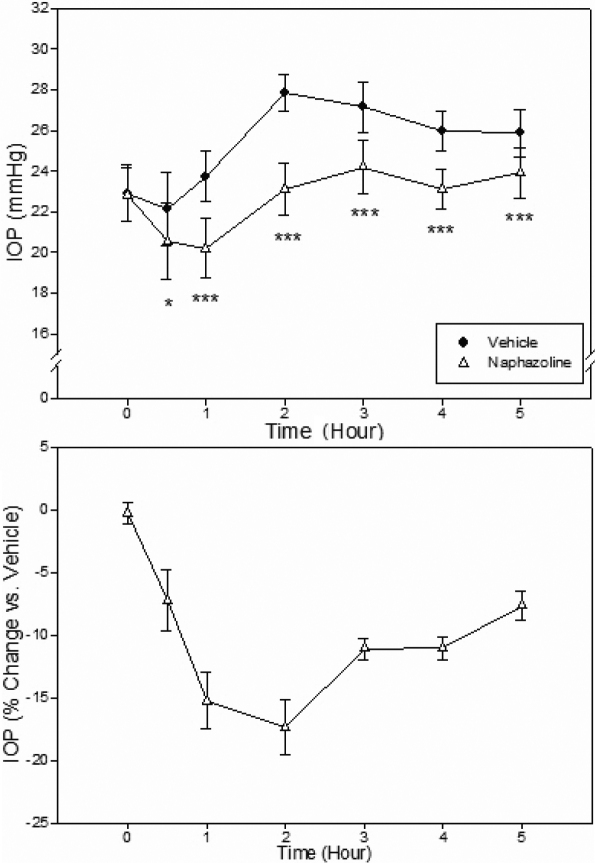
Effect of naphazoline (0.3%, 30 µl) on IOP of conscious Tibetan monkeys. The drug was administered topically at 10 AM (Time 0) onto a randomly assigned eye of each animal, while the contralateral eye received vehicle as control Error bars represent SEM (n=6). Top Panel: IOP values of both groups. *p<0.05, ***p<0.001 between the two treatment groups by two-tailed paired Student's *t*-test. Bottom panel: % IOP change of the naphazoline group relative to the vehicle control group.

**Figure 7:**
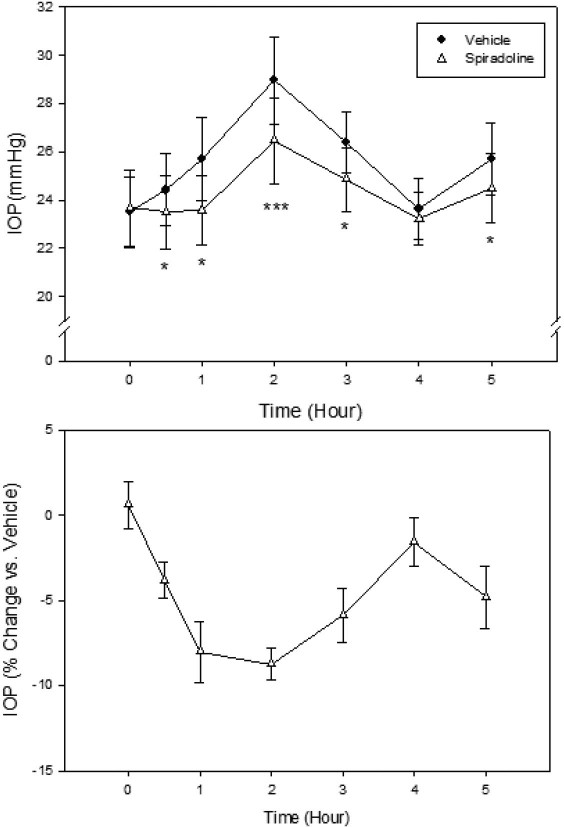
Effect of spiradoline (0.3%, 30 µl) on IOP of conscious Tibetan monkeys. The drug was administered topically at 10 AM (Time 0) onto a randomly assigned eye of each animal, while the contralateral eye received vehicle as control Error bars represent SEM (n=6). Top Panel: IOP values of both groups. *p<0.05, ***p<0.001 between the two treatment groups by two-tailed paired Student's *t*-test. Bottom panel: % IOP change of the spiradoline group relative to the vehicle control group.

Recently, compounds that increase aqueous concentrations of natriuretic peptides, such as naphazoline, an adrenergic α2 and imidazoline I1 receptor agonist, and spiradoline, a kappa opioid receptor agonist, were shown to reduce IOP in the rabbit [19,20]. We also evaluated them in the Tibetan monkeys. Both compounds significantly lowered monkey IOP, although naphazoline was clearly more efficacious. Topical ocular administration of naphazoline (100 μg; 0.33%, 30 µl) reduced monkey IOP maximally by 17.4% (4.8 mmHg; p<0.001) at 2 h after treatment. When compared to the contralateral vehicle-treated eye, a statistical significant IOP lowering lasted for at least 5 h (Figure 6). Spiradoline (100 µg; 0.33%, 30 µl) induced a maximum IOP reduction of 8.7% (2.5 mmHg; p<0.001) 2 h after drug administration (Figure 7).

## Discussion

In this study, we successfully trained young Tibetan monkeys to accept IOP measurement by the handheld TonoVet® rebound tonometer without sedation or anesthesia. According to our experience, the Tibetan monkeys, likely because of their tranquil and calm nature, are much easier to handle than rhesus and cynomolgus monkeys. Together with the extremely light touch of the rebound tonometer, it was possible to train them to allow IOP measurement under gentle restrain in a conscious state, seemingly without stress or discomfort. As indicated by the data reported here, the IOP values thus acquired have been consistent and reproducible.

In the trained young monkeys, we characterized their circadian IOP fluctuation and found that IOP was between 20 and 24 mmHg at most time points, but was typically higher during the diurnal phase and lower in the nocturnal phase. This circadian pattern of IOP changes does not correlate with that observed in human by Liu et al. [21,22] In the human studies, the nocturnal IOP was the highest. However, nocturnal IOP measurements in the human studies were performed with the subjects in a supine position, while the diurnal measurements were conducted with the subjects sitting. The change in postures confounds the results. Since the Tibetan monkeys usually sleep in a sitting/upright posture, and during IOP measurements in this study, they were held in an upright position, the issue related to supine IOP value does not apply and may explain the difference in results between this study and the human IOP studies. Regardless, our findings agree with previous reports of other monkeys [17]. Both young rhesus monkeys and cynomolgus monkeys were shown to have higher IOP in the morning and early afternoon [15,16,23]. Unique to our findings, we noticed a distinct peak of IOP at noon. We speculated that this may be a result of the short duration (approximately 30 min) between feeding and the assessment of IOP. Indeed, when the feeding time was moved from 11:30 AM to 12:30 PM, the peak IOP at noon was significantly diminished. Effect of eating on IOP has been reported previously [24]. However, the mechanism of this effect is currently unknown. It is possible that the animals were agitated during feeding, which stimulated certain components of the autonomic nervous system and in turn elevated IOP [25]. Clarification of this awaits further studies.

It is well known that general anesthesia lowers IOP in animals, including monkeys [15,23,26-28]. We confirmed these observations in the Tibetan monkeys. Interestingly, we found that the IOP-lowering effect of ketamine was prominent at the peak IOP measured at noon, but not apparent at the trough IOP recorded at 3 AM, likely because the IOP was already low at this time point. We also found that under anesthesia, young and mature monkeys did not differ significantly in their IOP, although the young monkeys had a slightly higher level. Regardless, general anesthesia confounds interpretation of the IOP data. Measurement on conscious animals avoids such undesirable effect and is thus clearly preferred.

Tibetan monkeys respond to clinically available ocular hypotensive medications similar to other primates. Travoprost is an ester prodrug of a potent FP prostaglandin receptor agonist, widely used as a treatment for glaucoma [29]. FP prostaglandin agonists, such as travoprost and latanoprost, are potent and efficacious IOP-lowering drugs in monkeys [29,30]. These compounds typically cause a reduction of IOP with a 2 to 4 h lag time after topical administration and the effect lasted for at least 24 h [16,31,32]. In the current study, we confirmed the prolonged IOP-lowering effect of travoprost in the Tibetan monkeys: the drug reduced IOP by approximately 20% for more than 24 h.

The beta-adrenergic receptor antagonist timolol was also effective in lowering IOP in the Tibetan monkeys. However, it was clearly less efficacious than travoprost; an IOP reduction of only 2.8 mmHg was seen. This result is similar to those reported in other monkey species. Although timolol lowers IOP by more than 9 mmHg in laser photocoagulation-induced ocular hypertensive monkeys [33], it typically produces rather small effects in normotensive monkey eyes. For example, in the normotensive cynomolgus monkey eyes, timolol only lowers IOP by approximately 2 mmHg [34]. In normotensive eyes of rhesus monkey, only a 2.6 mmHg reduction was reported [35]. Hence, regarding beta-blockers, the IOP response of the Tibetan monkey agrees with other primates.

In addition to confirming the IOP responses of these monkeys to known ocular hypotensive agents, we also tested effects of novel compounds. In the rabbit, naphazoline, an adrenergic α2 and imidazoline I1 receptor agonist, and spiradoline, a kappa opioid receptor agonist, were shown to increase natriuretic peptide levels in the aqueous humor [19,20]. Natriuretic peptides in ocular tissues elevate cellular levels of cyclic GMP, which has been demonstrated to lower IOP [36,37]. Indeed, both naphazoline and spiradoline lower rabbit IOP [19,20]. We confirmed that in the Tibetan monkeys, these compounds lower monkey IOP, with naphazoline being more efficacious. These preliminary findings support further careful evaluation of this pharmacological class of compounds for potential novel treatment of glaucoma.

Thus, we have demonstrated that after behavioral training, IOP measurement can be performed in conscious Tibetan monkeys. Their baseline IOP, circadian characteristics, and responses to several compounds are similar to other monkey species. Because of their tame, calm nature, these monkeys are easy to handle. Their relatively long life span among macaques allows a long-term, sustainable use for experiments, which reduces the costs of procurement and training. These advantages make them a viable alternative primate model for glaucoma research and evaluation of IOP-lowering compounds.
